# “Realistic strategies” and neutral processes drive the community assembly based on leaf functional traits in a subtropical evergreen broad‐leaved forest

**DOI:** 10.1002/ece3.9323

**Published:** 2022-09-17

**Authors:** Lijuan Zhao, Wenhua Xiang, Jiaxiang Li, Wenqian Liu, Yanting Hu, Huili Wu, Yiling Zhang, Xing Cheng, Weijia Wang, Wentao Wang, Shuai Ouyang

**Affiliations:** ^1^ Faculty of Life Science and Technology Central South University of Forestry and Technology Changsha China; ^2^ College of Forestry Central South University of Forestry & Technology Changsha China

**Keywords:** community assembly, environmental and spatial variables, leaf functional traits, phylogeny, subtropical evergreen broad‐leaved forest

## Abstract

*Neutral‐theory‐based stochastic* and *niche‐theory‐based determinative processes* are commonly used to explain the mechanisms of natural community assembly. However, considerable uncertainty remains regarding the relative importance of different ecological processes in shaping forest communities. Functional traits and phylogeny provide important information about plant environmental adaptation strategies and evolutionary history and promise a better mechanistic and predictive understanding of community assembly. Based on nine leaf functional traits and phylogenetic data of 18 dominant species in a *Lithocarpus glaber*–*Cyclobalanopsis glauca* evergreen broad‐leaved forest, we analyzed the variation in traits, explored the influence of phylogeny and environment on leaf traits, and distinguished the relative effects of spatial and environmental variables on functional traits and phylogenetic compositions. The results showed the following: (i) Leaf traits had moderate intraspecific variation, and significant interspecific variation existed especially among life forms. (ii) Significant phylogenetic signals were detected only in leaf thickness and leaf area. The correlations among traits both supported “the leaf economics spectrum” at the species and community levels, and the relationships significantly increased or only a little change after removing the phylogenetic influence, which showed a lack of consistency between the leaf functional trait patterns and phylogenetic patterns. We infer the coexistent species tended to adopt “realism” to adapt to their habitats. (iii) Soil total potassium and phosphorus content, altitude, aspect, and convexity were the most critical environmental factors affecting functional traits and phylogenetic composition. Total environmental and spatial variables explained 63.38% of the variation in functional trait composition and 47.96% of the variation in phylogenetic structures. Meanwhile, the contribution of pure spatial factors was significantly higher than that of the pure environment. *Stochastic processes* played dominant roles in driving community functional trait assembly, but *determinative processes* such as environmental filtering had a stronger effect on shaping community phylogenetic structure at a fine scale.

## Graphical abstract

We infer the coexistent species tended to adopt “realism” to adapt to their habitats.Neutral‐ theory‐based stochastic processes played dominant roles in driving community functional trait assembly, but niche‐theory‐based determinative processes such as environmental filtering had a stronger effect on shaping community phylogenetic structure at a fine scale.

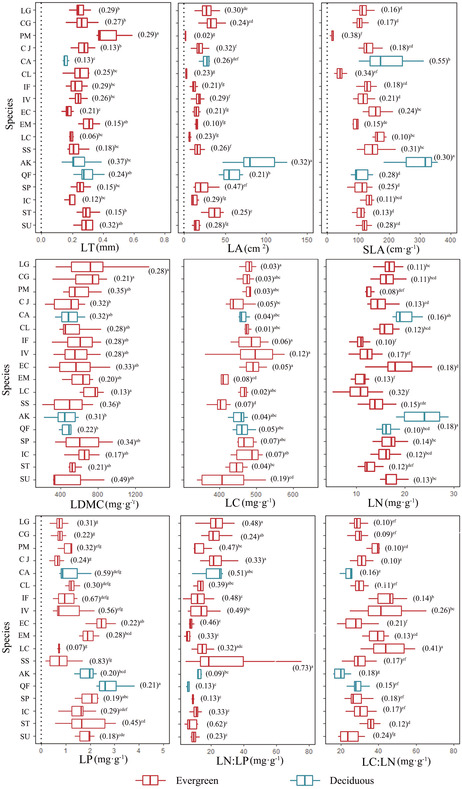

## 1 INTRODUCTION

Community assembly mechanisms have always been a topic of ecological research, and natural communities are generally believed to be structured by a set of processes (Chase, 2014; Levine et al., 2017; Wang et al., 2021). *Niche‐theory‐based determinative processes*, including the influence of the abiotic environment on fitness (Wang et al., 2021; e.g., habitat filtering) and biotic interactions, in particular interspecific competition (Leibold, 1998), and *neutral‐theory‐based stochastic processes*, including spatial dispersal limitation, demographic stochasticity, and ecological drift (Chase & Myers, 2011; Hubbell, 2005; Zhou & Zhang, 2008) have been regarded as two primary ecological mechanisms driving community assembly (Li et al., 2019). The relative importance of these processes tends to vary among ecosystems (Jiang et al., 2018; Liu et al., 2013), spatial scales (Zhang et al., 2021), community succession (Csecserits et al., 2021), and even in different environments, especially extreme environments (Wang et al., 2021). For example, deterministic processes may play a greater role than stochastic processes in adverse environments (Chase & Myers, 2011). Interspecific interactions and density‐dependent mechanisms should be strongest at the neighborhood scale where individual organisms interact, and environmental filtering should be stronger than interspecific interactions at the habitat scale (Cavender‐Bares et al., 2009; Purschke et al., 2017).

Plant functional traits are usually used as proxies to determine whether different tree species have different ecological strategies for resource capture and reproduction (Adler et al., 2013; Baraloto et al., 2012; Liu et al., 2020; McGill et al., 2006). Analyses of the distribution of trait values within communities yield insights of the ecological processes constraining their assembly (Kraft et al., 2008; Paine et al., 2011). If the niches of two species overlap, it is generally expected that the two species are similar in a range of functional traits, and vice versa (Westoby & Wright, 2006). Based on competition theory, higher similarity in functional traits for a community could lead to an increased intensity of interactions among neighboring individuals (Uriarte et al., 2004; Paine et al., 2011; Funk & Wolf, 2016). Consequently, communities with scattered trait values are primarily shaped by niche differentiation, whereas environmental filtering is the dominant process shaping ecological communities when trait value range is narrower than predicted (Paine et al., 2011). Thus, based on the fact that functional traits could represent the key aspects of physiology, investigating the variation in functional traits at the species level (i.e., intraspecific and interspecific variation) and at the community level could be beneficial for a deeper understanding of how physiological processes shape the assembly of ecological communities. Plant leaves are critical organs for the exchange of matter and energy with the photosynthetic organs of plants, and several biological processes such as plant growth, survival, reproduction, and ecosystem function are fully influenced by leaf parameters (e.g., leaf area, length, and dry mass; Surya et al., 2020). Leaf functional traits are sensitive to changes in environmental factors. They can adjust resource utilization strategies to adapt to different habitats through trade‐offs of various traits, which can reflect the driving mechanisms of the environment on community assembly (Tian et al., 2016; Wright et al., 2004; Zhang et al., 2020).

Although functional traits could provide a way to infer prevailing ecological process information based on morphological, physiological, and ecological characteristics, plant functional traits are not only affected by environmental factors but also by species evolution history (Swenson, 2013). Species coexisting in the same habitat might be relatives sharing common functional traits influenced by evolutionarily conserved or perhaps distant relatives adopting convergent traits to adapt to the habitat (Cavender‐Bares et al., 2009). Thus, phylogeny and functional traits do not necessarily present similar information and patterns (Cadotte et al., 2019), and testing the phylogenetic signals of functional traits is a necessary key step to more accurately infer the mechanism of community assembly (Baraloto et al., 2012; Cheng et al., 2019). Phylogeny is an indirect estimation of ecological similarity based on species affinity and an estimation of the impact of historical factors on the existing community (Jiang et al., 2018). Therefore, the combined analysis of phylogeny and functional traits can not only reveal the impact of community evolutionary history and functional traits simultaneously on the current community ecological process (Webb et al., 2002; Zhou et al., 2021), but also contribute to revealing the ecological processes responsible for evolution and functional assembly (Zhou et al., 2021). In other words, combining trait‐based and phylogenetic‐based approaches is a powerful way to detect community assembly processes (Gianuca et al., 2017; Kraft & Ackerly, 2010; Li et al., 2019).

Subtropical region of China holds the largest evergreen broad‐leaved forest in the world and harbors abundant seed plants and endemic species (Xu et al., 2017), which play an important role in biodiversity protection and carbon balance. However, due to the prolonged and frequent anthropogenic interferences, vegetation degradation is severe, and ecological problems are prominent in this area. Research based on the typical community structure and ecological processes in zonal vegetation has become an important means of vegetation restoration and reconstruction (Ouyang et al., 2016; Zhang et al., 2020; Zhao et al., 2015). As one of the typical types of evergreen broad‐leaved forests in the subtropical China, *Lithocarpus glaber*–*Cyclobalanopsis glauca* forests exhibit high species diversity, stable community structure, and high ecosystem function and service values (Zhao et al., 2015). To study the assembly processes and mechanisms, we carried out a series of studies focusing on species composition, spatial patterns, and effects of topographic and soil factors on woody species assembly in this long‐term monitoring community. We found that aggregation was the major spatial pattern and environment (topographic and soil factors) explained 28.10% of the species assembly (Zhao et al., 2015), and it is apparent that species‐based approaches to understand community assembly are limited. Here, we re‐examined this issue by integrating functional and phylogeny‐based approaches to explore the community assembly processes. The degree to which patterns of functional traits and phylogenetic dispersion may be easily explained by the abiotic environment and spatial relationship has been also discussed. We propose that a variance partitioning approach can be applied to simultaneously address these challenges (Zhang et al., 2021). The degree to which the abiotic environment, dispersal limitation, and/or their joint effects affect trait dispersion should be determined by partitioning variance in trait dispersion into pure environmental, spatial, and joint effects. We also measured nine leaf functional traits and phylogenetic data of 18 dominant woody species with important value >1.00% and accurate topographic and edaphic data sets to address the following: (i) to clarify the variation and trade‐off relationship of leaf functional traits; (ii) to explore the effects of phylogeny and environment on trait variation; (iii) to disentangle the relative importance of niche and neutral processes shaping community assembly at a fine scale.

## 2 MATERIALS AND METHODS

### 2.1 Study site and species selection

**FIGURE 1:**
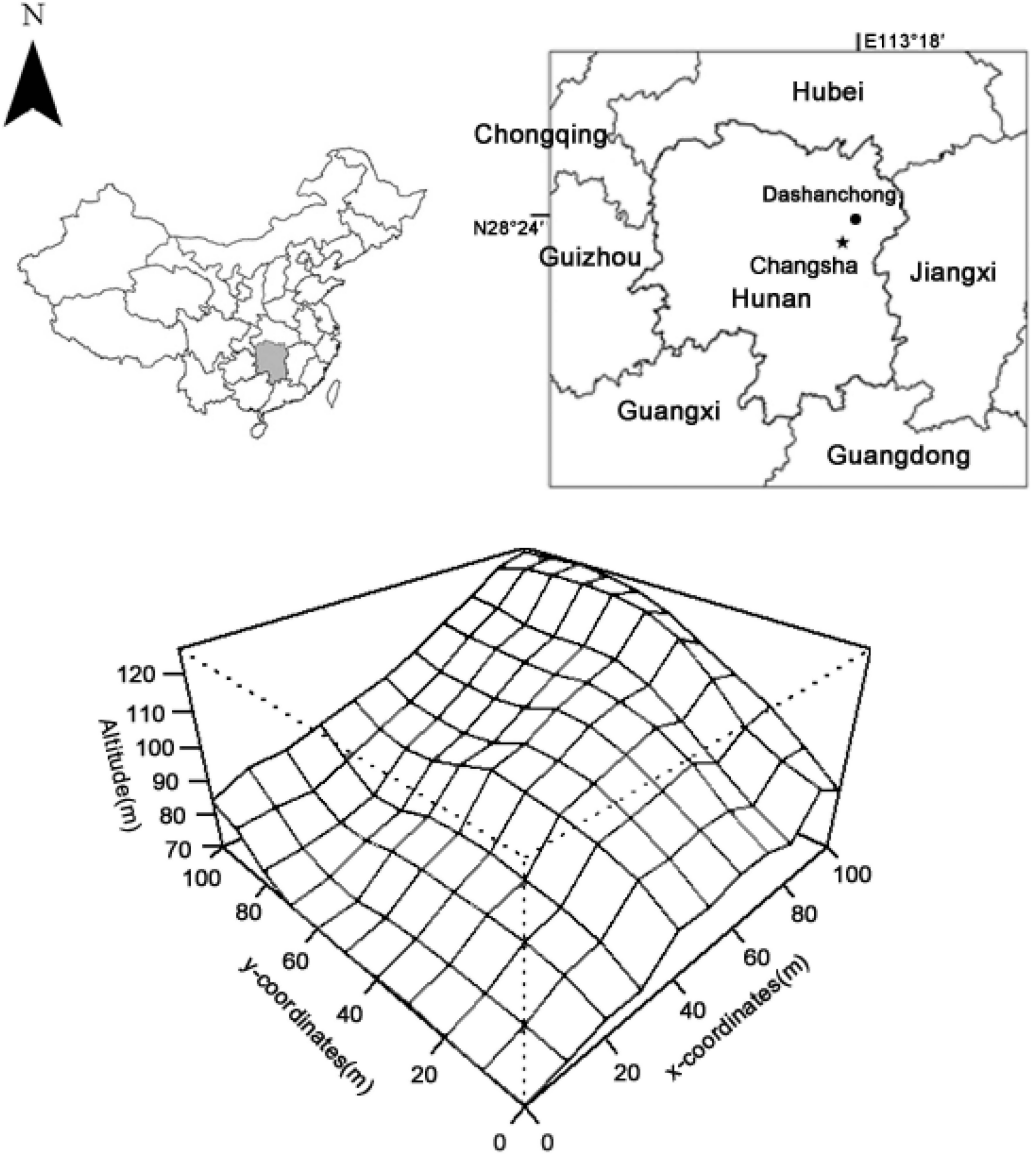
Location and map of the 1‐ha permanent plot of *Lithocarpus glaber*–*Cyclobalanopsis glauca* broad‐leaved evergreen forest at Dashanchong Forest Farm, Changsha County, Hunan Province, China.

This study was conducted in the Dashanchong National Forest Park (28°23′58″–28°24′58″N, 113°17′46″–113°19′8″E), Changsha County, Hunan Province, China (Figure 1). This region is typical of a low hilly landscape 55–217.40 m above sea level. The climate is humid mid‐subtropical monsoon climate with an annual mean temperature of 17.30°C and mean monthly temperatures of −10.30°C in the coolest month (January) and 39.80°C in the warmest month (July). The mean annual precipitation is 1416 mm (Ouyang et al., 2016; Wu et al., 2019). The soil type was well‐drained clay loam red soil developed on slate and shale rocks. The regional climax vegetation was subtropical evergreen broad‐leaved forest. *L. glaber*–*C. glauca* forest, a well‐preserved evergreen broad‐leaved forest, is a multi‐dominant, unevenly aged forest with structural stability (Zhao et al., 2015) that represents the succession direction of vegetation in the subtropical hilly region.

One‐hectare permanent plot (100 × 100 m horizontal distance, Figure 1) of *L. glaber*–*C. glauca* evergreen broad‐leaved forest was established in 2009. The plot was divided into 100 10 × 10 m subplots to conduct a census. The second survey and leaf sampling were conducted in 2019. In this study, 18 dominant species with importance values ≥1.00% measured as the method of Zhao et al. (2015) were examined accounting for 89.38% (Table 1).

**TABLE 1:** Nomenclature and important values of 18 dominant woody species in *L. glaber*–*C. glauca* evergreen broad‐leaved forest community.

Species	Abbreviation	Family	Life form	Important value (%)
*Lithocarpus glaber*	LG	Fagaceae	Evergreen	21.78
*Cyclobalanopsis glauca*	CG	Fagaceae	Evergreen	12.02
*Pinus massoniana*	PM	Pinaceae	Evergreen	10.60
*Cleyera japonica*	CJ	Pentaphylacaceae	Evergreen	9.33
*Choerospondias axillaris*	CA	Anacardiaceae	Deciduous	6.29
*Cunninghamia lanceolata*	CL	Taxodiaceae	Evergreen	4.45
*Ilex formosana*	IF	Aquifoliaceae	Evergreen	3.95
*Ilex viridis*	IV	Aquifoliaceae	Evergreen	3.88
*Elaeocarpus chinensis*	EC	Elaeocarpaceae	Evergreen	2.86
*Eurya muricata*	EM	Pentaphylacaceae	Evergreen	2.34
*Loropetalum chinense*	LC	Hamamelidaceae	Evergreen	2.15
*Symplocos setchuensis*	SS	Symplocaceae	Evergreen	2.06
*Alangium kurzii*	AK	Cornaceae	Deciduous	1.85
*Quercus fabri*	QF	Fagaceae	Deciduous	1.51
*Symplocos pendula* var. *hirtistylis*	SP	Symplocaceae	Evergreen	1.48
*Ilex chinensis*	IC	Aquifoliaceae	Evergreen	1.12
*Symplocos stellaris*	ST	Symplocaceae	Evergreen	1.01
*Symplocos sumuntia*	SU	Symplocaceae	Evergreen	1.00
Total				89.38%

### 2.2 Leaf sample collection and functional traits measurement

**FIGURE 2:**
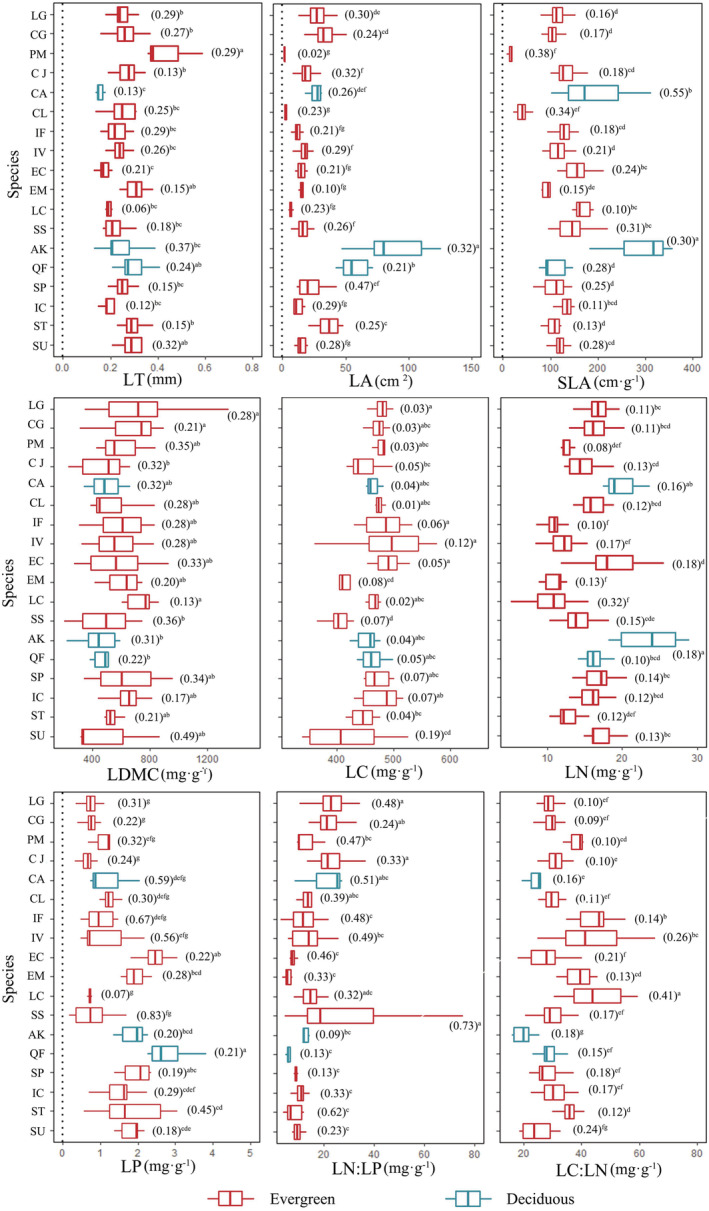
Intraspecific and interspecific variations in leaf functional traits of the 18 dominant species. LT, LA, SLA, LDMC, LC, LN, LP, LN:LP, and LC:LN represent leaf thickness, leaf area, specific leaf area, leaf dry matter content, leaf carbon content, leaf nitrogen content, leaf phosphorus content, leaf nitrogen‐phosphorus ratio, and leaf carbon‐nitrogen ratio, respectively. Different letters and their combinations represent the significance levels of interspecific differences (*p* < .05). The numbers in brackets indicate the intraspecific variation coefficients of each species. See Table 1 for species abbreviation codes.

For each leaf trait, 10 replicates were obtained from individuals of three standard trees of each dominant species (Pérez‐Harguindeguy et al., 2013) growing on a 10 m × 10 m subplot. Fully expanded sun leaves above the crown were collected and packed in sealed moist plastic bags with damp paper that were transported to the laboratory in a cooler with ice to preserve the water saturation of the leaves until the time of the measurements. We measured the following structural functional traits: leaf thickness (LT, mm), leaf area (LA, mm^2^), specific leaf area (SLA, cm^2^ g^−1^), leaf dry matter content (LDMC, mg g^−1^), and chemical functional traits: leaf carbon (LC, mg g^−1^), leaf nitrogen (LN, mg g^−1^), and leaf phosphorus (LP, mg g^−1^) contents; leaf nitrogen: phosphorus ratio LN:LP and leaf carbon: nitrogen ratio (LC:LN; Figure 2).

We used different methods to measure the leaf thickness of the broad‐leaved and coniferous trees. For broad‐leaf species, thickness values were measured at five points per leaf from the front, middle, and end with digital caliper (Dasqua 150 mm Special Glass Grating Big Screen Digital Caliper, Sichuan, China, accurate to 0.02/0.001 mm), avoiding the mid‐vein and secondary veins to reduce sampling variation, and then the average value was taken as LT. For conifer species, LT was obtained from the middle of the needles (He et al., 2020). The blades were then laid on A4 papers, flattened with a transparent plastic sheet to ensure that each blade was fully unfolded, and scanned to obtain a plane image. Adobe Photoshop CS 6 software (Xiao et al., 2005) was used to calculate the pixel points occupied by each leaf, and the average value was taken as the LA of broad‐leaf species. For conifer species, leaf length (LL) and middle leaf width (LW) were determined to calculate as LA = (π × LL × LW)/2.

Ten leaves per species were randomly selected and soaked in deionized water for 12 h in the dark. After removing the leaves, the excess water was immediately absorbed, and the saturated fresh weight (g) was weighed using an electronic balance (AR2140, OHAUS, Guangzhou Jingbo Electronics Co., Ltd，accurate to 0.0001). The leaves were then oven‐dried to a constant weight at 65°C for at least 72 h and then weighed using an electronic balance. Leaf area and dry mass (g) were then used to calculate the leaf dry matter content (LDMC = leaf dry weight/saturated fresh weight) and specific leaf area (SLA = leaf area/leaf dry weight; Yang et al., 2021).

After measuring structural traits of leaf, we oven‐dried the leaves at 105°C for 5 min to eliminate the green and then dried them again at 85°C to a constant weight. The leaves were milled and sieved through a 0.15 mm mesh. LC, LN, and LP were measured using the potassium dichromate heating, Kjeldahl, and molybdenum‐antimony colorimetric methods, respectively (Wu et al., 2019). Two sets of parallel experiments and two blank tests were performed simultaneously for all parameter measurements to reduce the experimental errors. The average value was taken as the value of the parameter of the subplot and species and was considered to calculate the LN:LP and LC:LN.

### 2.3 Variables used in redundancy analysis (RDA) and variation partitioning

Environmental and spatial variables were generated as descriptors, and functional and phylogenetic compositions were generated as response variables before RDA and variation partitioning. Four topographic variables (mean altitude, slope, convexity, and aspect) and seven soil variables (mean soil moisture; pH; and concentrations of total carbon (TC), total nitrogen (TN), total phosphorus (TP), total potassium (TK), and available phosphorus (AP)) were measured as environmental variables. All topographic variables were calculated based on the elevations at the four corners of each subplot. Edaphic variables were measured using systematic and random sampling approaches to collect soil samples near the central point of each subplot. Detailed descriptions and formulas of the topographic and edaphic sampling methods can be found in the study by Zhao et al. (2015).

Principal coordinates of neighbor matrix (PCNM) eigenvectors were generated to describe the spatial variables and were then used as indirect proxies of dispersal‐based processes (Borcard & Legendre, 2002). First, we used the central coordinates (X and Y) of each subplot to derive the Euclidean distance matrix. Second, a truncated matrix was generated by retaining and replacing the values in the distance matrix using a threshold (Jiang et al., 2018). Finally, the PCNM variables were generated by performing principal coordinate analysis on the truncated distance matrix (Dray et al., 2012). A total of 57 PCNM variables with eigenvalues higher than zero were generated as spatial variables at a fine spatial scale (10 m × 10 m) using the PCNM function in the vegan package (Oksanen et al., 2013).

To compare the effects of different response variables on the detection of ecological processes, we used 11 response variables, including multivariable, single functional, and phylogenetic variables. First, we calculated the community‐weighted mean (CWM; Bruelheide et al., 2018) taking the relative importance value of tree species within each subplot as the weight. The formula used is as follows: Trait_*c*_ = Σ*P*_*i*_ × trait_*i*_, where Trait_*c*_ represents the CWM of *i* functional trait of leaf. Trait_*i*_ and *P*_*i*_ are the values of functional traits of each species and the important value for species *i* in a subplot. Second, we used phylogenetic dendrograms and employed the phylogenetic fuzzy weighting (PFW) method to generate phylogenetic compositions (Jiang et al., 2018). Detailed descriptions of the calculations and background of the PFW approach can be found in the study by Duarte et al. (2016). This analysis was conducted using principal coordinates of phylogenetic structure (PCPS; Debastiani & Duarte, 2014). Before analysis, all environmental variables (except convexity) and CWM of traits were log‐transformed, and phylogenetic compositions were Hellinger‐transformed.

### 2.4 Statistical analysis

To test the intraspecific and interspecific variations in leaf functional traits, we performed descriptive statistics on the leaf trait data of each species, calculated the mean, range, standard deviation (SD), standard error (*SE*), coefficient of variation (CV), and skewness of each trait and then tested the difference of each trait using the multiple comparison method of least significant difference (LSD).

To detect the phylogenetic signals of traits, phylogenetic independence analysis (phylogenetic independent contrasts, PICs) was performed and a phylogenetic dendrogram combined with functional traits was constructed. The species details (family/genus/species) were input into Phylomatic (http://www.phylodiversity.net/phylomatic) to construct a phylogenetic tree. For this, the Angiosperm Phylogeny Group's APG III consensus tree was taken as the pedigree skeleton and evolutionary branch length using Figtree was obtained (Webb et al., 2008). Next, we used Blomberg's *K* test, which compares the variance of PICs with that expected in a Brownian motion (random) model (Blomberg et al., 2003). This analysis implemented the “phylosignal” function in the “picante” package (Kembel et al., 2010) and the phylogenetic tree of the species with 999 randomizations. A significant *p* value (<.05) in this analysis indicates that a phylogenetic signal exists and that phylogenetically close species are more similar than random pairs of species.

To explore the relationships among the nine traits, we determined correlations existing between traits and trait CWMs. The Pearson's correlation coefficients among leaf traits at the species and community levels were determined. Then, we examined the species‐level trait relationships using PICs and community‐level trait relationships using partial regression analysis by controlling the phylogenetic variables.

Further, RDA analysis was used to explore the influence of topography and soil on leaf functional trait and phylogenetic compositions at community level using the vegan package (Oksanen et al., 2013). To distinguish the effect of stochastic and niche processes on community assembly, variation partitioning was used to assess the relative importance of environmental and spatial variables as explanatory variables for the variations of functional traits (CWMs) and phylogenetic compositions using the rdacca.hp function in the rdacca.hp package (Lai et al., 2022).

All analyses were performed using R version 3.6.0 (R Development Core Team, 2016).

## 3 RESULTS

### 3.1 Variations in leaf functional traits

The intraspecific variation in majority of the leaf functional traits was moderate, with a coefficient of variation (CV) ranging from 0.10 to 0.30 (Figure 2). The variation in the structural traits was relatively less, CV ranging from 0.02 to 0.55. Among the chemical traits, LC exhibited the least variation, while LP (CV = 0.07–0.83) and LN:LP (CV = 0.09–0.73) showed the greatest variations (Figure 2).

In contrast, leaf functional traits varied significantly among the species (*p* < .05). *Pinus massoniana* had the highest LT and the lowest LA and SLA. *Alangium kurzii* and *Quercus fabri* had higher LA, SLA, and LN but lower LDMC and LC:LN. The LN of the evergreen species was significantly lower than that of the deciduous and coniferous species (*P. massoniana* and *Cunninghamia lanceolata*). Deciduous species had higher LA, SLA, LN, and LP values than those of both evergreen and coniferous species. Coniferous species had the lowest LA and SLA (*p* < .05) but the highest LT (Figure 2).

At the community level, the variations in most of the traits were moderate (CV = 0.15–0.36), except LC_C_, LN_C_, and LC:LN_C_, where the CV <0.10 (Table 2). Among the structural characters, the greatest variation was seen in LA_C_ (CV = 0.36) ranging from 7.57 to 57.37 cm^2^. Among the chemical traits, LP varied greatly, especially LN:LP_C_ was 20.49 and the values ranged from 10.39 to 46.35 (CV = 0.24; Table 2).

**TABLE 2:** Descriptive statistics of the community‐weighted leaf functional traits based on important values of each species.

Traits	Mean	*SE*	Minimum	Maximum	CV	Skewness
LT_C_ (mm)	0.27	0.00	0.19	0.40	0.15	1.14
LA_C_ (cm^2^)	22.83	0.86	7.57	57.37	0.36	1.07
SLA_C_ (cm^2^·g^−1^)	116.40	3.27	61.27	213.03	0.27	1.17
LDMC_C_ (mg·g^−1^)	608.55	13.16	379.06	1088.17	0.21	0.66
LC_C_ (mg·g^−1^)	470.46	1.25	433.63	498.51	0.03	−0.45
LN_C_ (mg·g^−1^)	15.88	0.15	11.53	19.47	0.09	0.07
LP_C_ (mg·g^−1^)	0.94	0.02	0.47	1.67	0.21	0.50
LN:LP_C_	20.49	0.53	10.39	46.35	0.24	2.04
LC:LN_C_	30.97	0.30	25.22	40.99	0.09	0.43

*Note*: LT_C_, LA_C_, SLA_C_, LDMC_C_, LN_C_, LC_C_, LP_C_, LN:LP_C_, and LC:LN_C_ represent leaf thickness, leaf area, specific leaf area, leaf dry matter content, leaf nitrogen content, leaf carbon content, leaf phosphorus content, leaf nitrogen‐phosphorus ratio, and leaf carbon‐nitrogen ratio at the community level, respectively.

Abbreviations: CV, Coefficient of variation; *SE*, Standard error.

### 3.2 Phylogenetic signals and correlations of leaf functional traits

**FIGURE 3:**
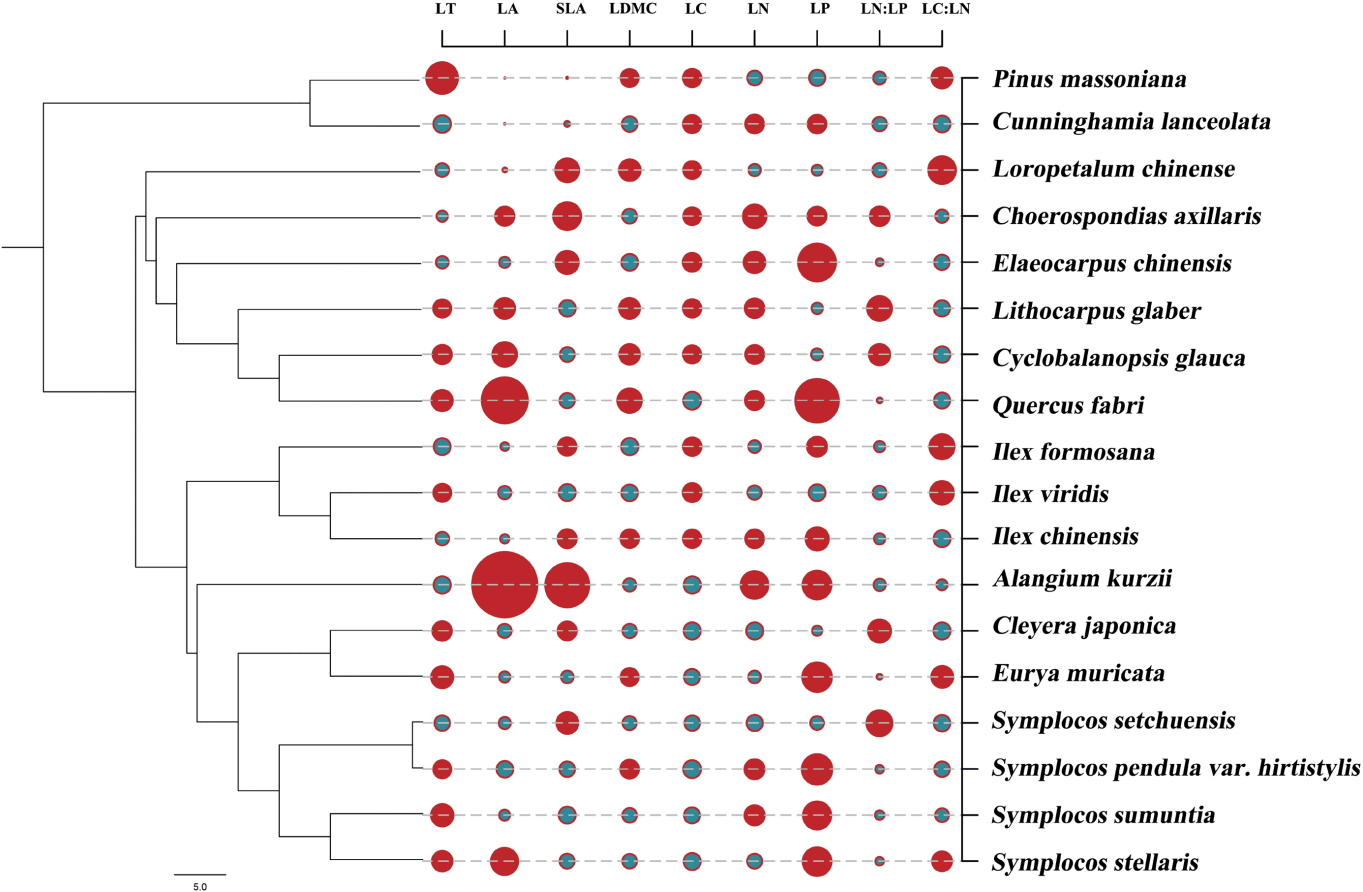
Functional dendrogram of the 18 dominant species in *Lithocarpus glaber*–*Cyclobalanopsis glauca* evergreen broad‐leaved forest community. LT, LA, SLA, LDMC, LC, LN, LP, LN:LP, and LC:LN represent leaf thickness, leaf area, specific leaf area, leaf dry matter content, leaf carbon content, leaf nitrogen content, leaf phosphorus content, leaf nitrogen‐phosphorus ratio, and leaf carbon‐nitrogen ratio, respectively. Symbol size indicates the proportion (%) of functional trait values for each species, with smaller symbols closer to the mean value; red symbols represent values above the mean, and green symbols represent values below the mean.

The functional dendrogram (Figure 3) showed that the leaf functional traits of the 18 coexisting woody species were phylogenetically structured, and species of the same genus clustered and had similar functional traits. Significant phylogenetic signals were detected only for LT and LA (*K* > 1, *p* < .01) among different species. The SLA showed a moderate phylogenetic signal (*K* = 0.53, *p* < .05). However, the LDMC and chemical traits (*K* = 0.05–0.40) were randomly distributed in the phylogeny because there were no significant phylogenetic signals (*p* < .05, Table 3).

**TABLE 3:** Phylogenetic signals of leaf functional traits for all species.

Traits	*K*	*p* Value
LT (mm)	1.5	**.002**
LA (cm^2^)	1.05	**.002**
SLA (cm^2^·g^−1^)	0.53	**.033**
LDMC (mg·g^−1^)	0.4	.107
LC (mg·g^−1^)	0.25	.298
LN (mg·g^−1^)	0.2	.374
LP (mg·g^−1^)	0.05	.949
LN:LP	0.06	.922
LC:LN	0.19	.398

*Note*: LT, LA, SLA, LDMC, LC, LN, LP, LN:LP, and LC:LN represent leaf thickness, leaf area, specific leaf area, leaf dry matter content, leaf carbon content, leaf nitrogen content, leaf phosphorus content, leaf nitrogen‐phosphorus ratio, and leaf carbon‐nitrogen ratio, respectively. *K*: Blomberg's *K* values, and significant values at *p* < .005 are shown in bold. All the trait data were log‐transformed. *p*‐values for traits with significant phylogenetic signals are highlighted in bold.

At the species level, irrespective of phylogenetic influence, there were significant negative correlations between SLA with LT, LDMC, and LC:LN; and LN with LT. There was a clear positive relationship between SLA with LA, LN; and LN, LP with LA, respectively (Table 4). Interestingly, after removing the influence of phylogeny, the correlations between SLA with LT, LN; and LN with LA, LC; and LC with LT, LDMC; and LP with LC, LN were significantly enhanced (Table 4). At the community level, non‐phylogenetic correlations based on CWMs of the nine leaf traits were largely consistent with these results (Table 5). However, after controlling for phylogenetic variables, most of the relationships showed a little weakening trend, and only the correlations between SLA_C_ with LC_C_; and LP_C_ with LA_C_, LT_C_ increased slightly (Table 5).

**TABLE 4:** Pearson's correlation coefficients (lower diagonal) and phylogenetically independent contrasts (PIC, upper diagonal) among nine leaf functional traits for all species.

Traits	LT (mm)	LA (cm^2^)	SLA (cm^2^·g^−1^)	LDMC (mg·g^−1^)	LC (mg·g^−1^)	LN (mg·g^−1^)	LP (mg·g^−1^)	LN:LP	LC:LN
LT (mm)	1	−0.02	−0.65**	−0.40	−0.74***	−0.65**	−0.63**	−0.11	0.16
LA (cm^2^)	−0.08	1	0.50*	−0.14	−0.01	0.48*	0.35	−0.14	−0.43
SLA (cm^2^·g^−1^)	−0.49***	0.53***	1	−0.02	0.33	0.71**	0.40	0.13	−0.28
LDMC (mg·g^−1^)	0.06	−0.09	−0.32***	1	0.72***	0.12	0.52*	−0.14	0.27
LC (mg·g^−1^)	−0.10	−0.05	−0.09	0.17	1	0.49*	0.77***	−0.34	0.09
LN (mg·g^−1^)	−0.21***	0.53***	0.52***	−0.14	0.02	1	0.66***	0.08	−0.75***
LP (mg·g^−1^)	−0.10	0.27***	0.17	−0.10	−0.02	0.23***	1	−0.47	−0.26
LN:LP	0.01	0.01	0.04	−0.02	−0.07	0.20***	−0.70***	1	−0.29
LC:LN	0.08	−0.39***	−0.27***	0.10	0.29***	−0.85***	−0.17	−0.26***	1

*Note*: LT, LA, SLA, LDMC, LC, LN, LP, LN:LP, and LC:LN represent leaf thickness, leaf area, specific leaf area, leaf dry matter content, leaf carbon content, leaf nitrogen content, leaf phosphorus content, leaf nitrogen‐phosphorus ratio, and leaf carbon‐nitrogen ratio, respectively. ***p < .001; **p < .01; *p < .05.

**TABLE 5:** Pearson's correlation coefficients (lower diagonal) and phylogenetically independent contrasts (PIC, upper diagonal) among the nine leaf functional traits at the community level.

Traits	LT_c_ (mm)	LA_c_ (cm^2^)	SLA_c_ (cm^2^·g^−1^)	LDMC_c_ (mg·g^−1^)	LC_c_ (mg·g^−1^)	LN_c_ (mg·g^−1^)	LP_c_ (mg·g^−1^)	LN:LP_c_	LC:LN_c_
LT_c_ (mm)	1	−0.21	−0.62***	−0.03	0.13	−0.37**	−0.39***	0.30*	0.30*
LA_c_ (cm^2^)	−0.22*	1	0.25	0.14	−0.10	0.38**	0.31*	−0.13	−0.41**
SLA_c_ (cm^2^·g^−1^)	−0.62***	0.26**	1	−0.25	−0.37**	0.43**	0.22	−0.15	−0.35*
LDMC_c_ (mg·g^−1^)	−0.02	0.13	−0.26**	1	0.30*	−0.07	0.03	−0.10	0.11
LC_c_ (mg·g^−1^)	0.11	−0.08	−0.35***	0.29**	1	0.18	−0.20	0.28*	0.08
LN_c_ (mg·g^−1^)	−0.38***	0.39***	0.44***	−0.08	0.19	1	0.08	0.22	−0.91***
LP_c_ (mg·g^−1^)	−0.37***	0.28**	0.20	0.04	−0.21*	0.05	1	−0.72***	−0.06
LN:LP_c_	0.28**	−0.11	−0.14	−0.11	0.29***	0.23*	−0.72***	1	−0.21
LC:LN_c_	0.30***	−0.41***	−0.35***	0.12	0.07	−0.91***	−0.05	−0.22*	1

*Note*: LT_C_, LA_C_, SLA_C_, LDMC_C_, LN_C_, LC_C_, LP_C_, LN:LP_C_, and LC:LN_C_ represent leaf thickness, leaf area, specific leaf area, leaf dry matter content, leaf nitrogen content, leaf carbon content, leaf phosphorus content, leaf nitrogen‐phosphorus ratio, and leaf carbon‐nitrogen ratio at the community level, respectively. ***p < .001; **p < .01; *p < .05.

### 3.3 Primary environmental factors affecting leaf functional traits

**FIGURE 4:**
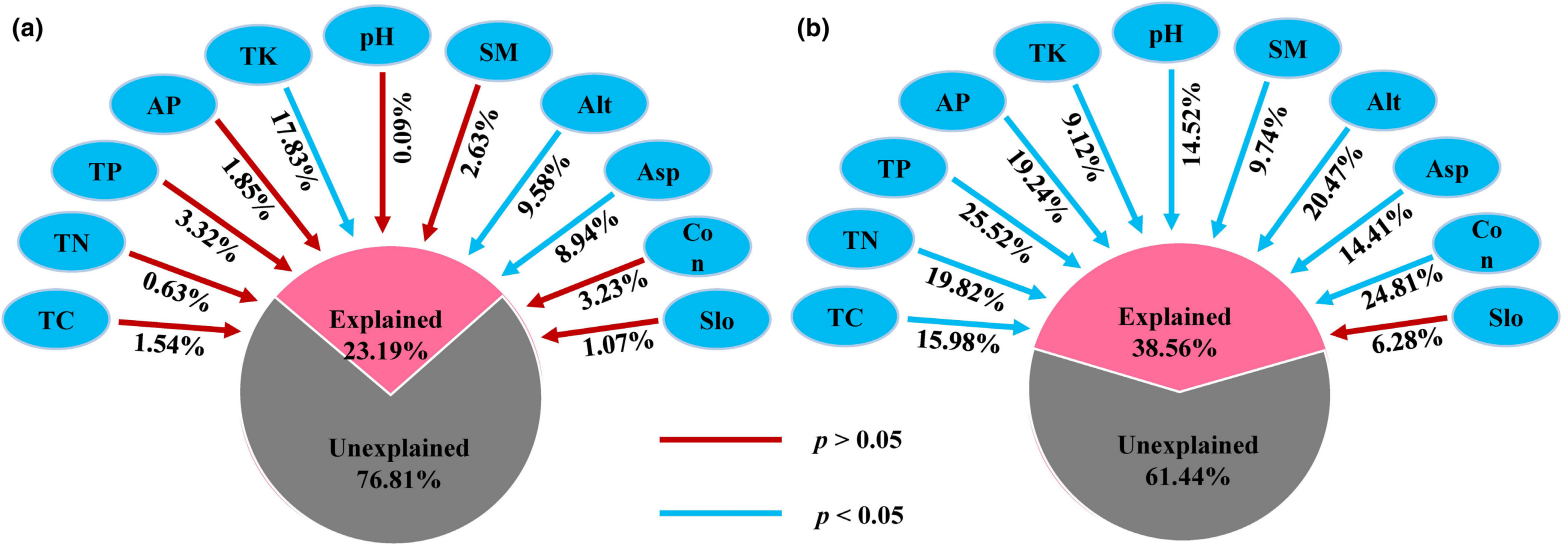
Effects of topographic and edaphic variables on leaf functional traits and phylogenetic compositions at the community level. TP, TC, TN, TK: total soil phosphorus, nitrogen, carbon, and potassium, respectively; AP—available phosphorus. Alt, Asp, Con, Slo: altitude, aspect, convexity, slope, respectively. (a) The effect of each variable (topography or soil) on community‐weighted mean (CWMs) of leaf functional traits. (b) The effect of each variable (topography or soil) on phylogenetic compositions.

At the community level, 23.19% of the variation in leaf functional traits was explained by environmental factors. TK was a unique significant edaphic factor (*R* ^2^ = 17.83%), and altitude and aspect were the two significant topographical factors explaining 9.58% and 8.94% of the variation, respectively (Figure 4a). For phylogenetic compositions, all factors showed significant effects except slope, which together explained 38.56% of the total variation. Among them, the most important environmental factors were TP, altitude, and convexity, each with an interpretation rate of over 20% (Figure 4b).

### 3.4 Environmental and spatial effects on functional traits and phylogenetic compositions

**FIGURE 5:**
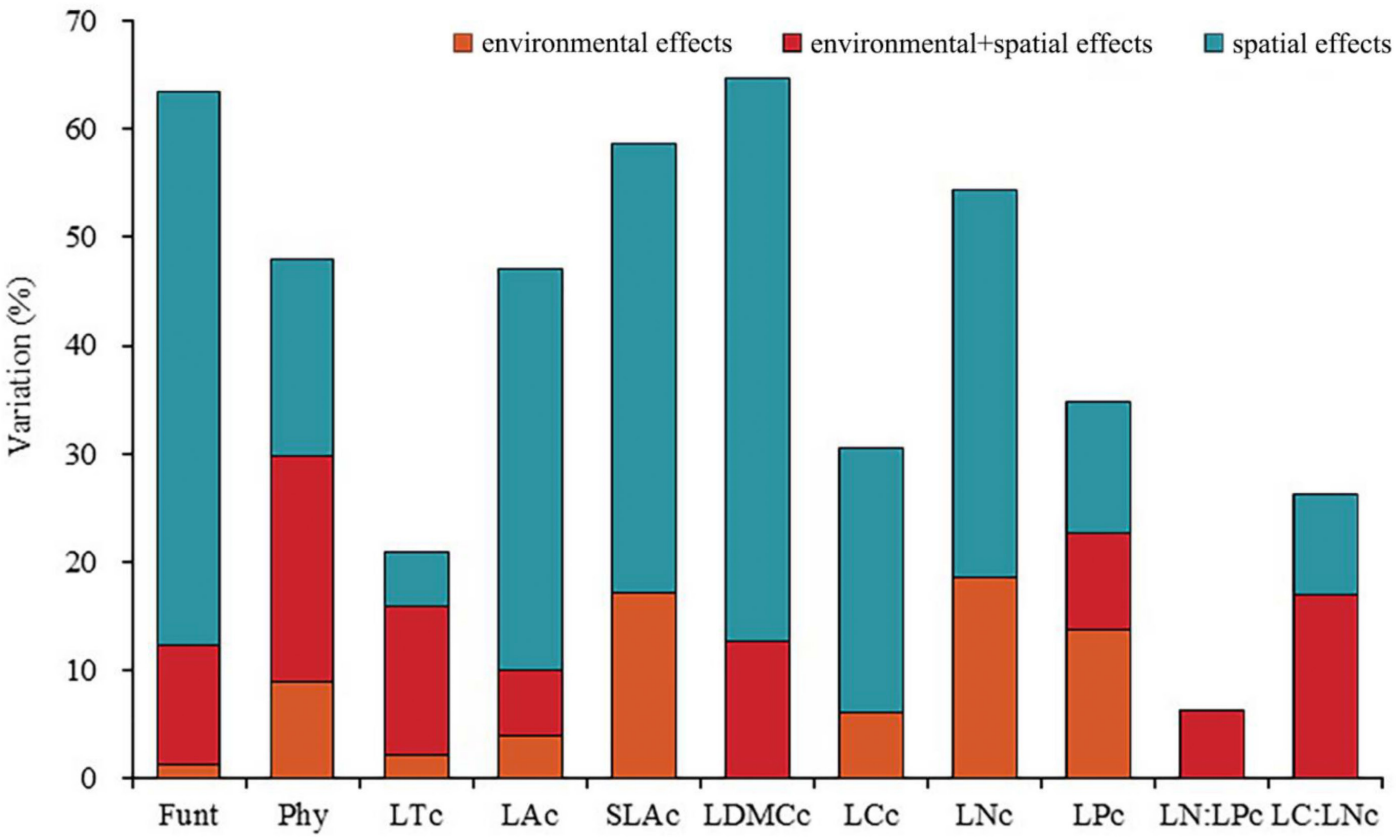
Variation partitioning of phylogenetic and functional traits structures (adjusted *R* ^2^ × 100%) in the effects of different response variables at the community level. The interpretation rates of purely environmental, shared environmental and spatial and purely spatial fractions on different response variable are indicated by orange, red, and blue, respectively. Response variables are the all community‐level functional traits (Funt_c_), phylogenetic (Phy_c_) structures and each community‐level trait composition, and LT_C_, LA_C_, SLA_C_, LDMC_C_, LN_C_, LC_C_, LP_C_, LN:LP_C_, and LC:LN_C_ represent leaf thickness, leaf area, specific leaf area, leaf dry matter content, leaf nitrogen content, leaf carbon content, leaf phosphorus content, leaf nitrogen‐phosphorus ratio, and leaf carbon‐nitrogen ratio at the community level, respectively.

The results of variation partitioning showed that the pure spatial factors (C) explained a higher variation in each functional trait at community level than pure environmental factors (A), except for LP_c_ and LN:LP_c_ (Figure 5). Environmental and spatial factors comprehensively accounted for 63.38% of the total variation in all community‐level functional traits and 47.96% of the phylogenetic structures. Meanwhile, the contribution of the pure environment and the combined effect of environment and space (B) to the phylogenetic structure were both significantly higher than those of the community‐level functional traits. However, for each community‐level trait, pure spatial variables explained 52.06%, 41.56%, and 37.07% variations of the LDMC_c_, SLA_c_, and LC_c_, respectively. The pure environment could explain 18.48% of the variation in LN_c_ at most, whereas it showed no influence on LC_c_, LN:LP_c_, and LC:LN_c_. The combined effect of environment and space on LT_c_ was the highest (13.75%), but it had no effect on LC_c_ and LN_c_ (Figure 5).

## 4 DISCUSSION

### 4.1 Variation in leaf functional traits and trade‐off relationships

We found that the mean values of nine leaf functional traits of 18 dominant tree species in this community were within the ranges of global leaf trait variation (Chen et al., 2016; Zhao et al., 2020), and it was moderate in intraspecific and community‐level variations, while significant in interspecific variation, indicating that accurate measurements of multi‐source variation of functional traits were significant for deep understanding of the processes of community assembly (Westerband et al., 2021). Compared to that in other vegetation types (Li et al., 2017; Liu et al., 2020; Wang et al., 2021), the intraspecific variation in this community was at a lower level and supported the “a spatial trait variance partitioning hypothesis,” that is, due to the limitation of environmental heterogeneity and individual number, the importance of intraspecific variation was less at the fine scale.

The functional traits of plant leaves are determined by both genetic and environmental factors. They usually show niche differentiation and divergence of ecological strategies through intraspecific variation so as to reduce the intensity of competition (Kang et al., 2017) and to adapt to a broad environmental gradient. In this community, the intraspecific variation in leaf chemical characteristics (LC, LN, LP, and LC:LN) of dominant tree species was higher than that of structural characteristics (LA, SLA, and LDMC). However, the interspecific variations showed the opposite trend except for the related‐LP traits, indicating that the variation in leaf chemical traits had higher plasticity in coping with local environmental changes. Zhao et al. (2015) found that the soil and topographic variables in the community had moderate spatial variation, which had significant effects on the species composition and spatial distribution of the community. And as we all know, the availability of nitrogen and phosphorus in soil is the main influencing factor influencing LN and LP contents, respectively (Li et al., 2017). Therefore, to adapt to more complex environmental conditions, dominant species increase their distribution space along the environmental gradient by adjusting the plasticity of leaf chemical traits. Compared with chemical traits, leaf structural traits are mainly limited by genetic factors and are relatively stable and closely related to life forms and species type; therefore, interspecific variation is larger (Ackerly & Reich, 1999). In addition, it greatly differs for species at different phylogenetic stages in leaf size, leaf life span, LN content, and photosynthetic ability. Eighteen dominant species in this community belonging to 10 families, including Fagaceae, Theaceae, Anacardiaceae, Pinaceae, and Taxodiaceae (Table 1), exhibited significantly different phylogeny background and thus may help them coexist through the coordination of functional traits. In addition, needle‐leaf trees are generally thought to be better adapted to cold or nutrient‐poor environments than broad‐leaf trees (Liu et al., 2020; Liu, Chen, et al., 2021; Liu, Li, et al., 2021). With an increase in water stress, plants tend to exhibit xerophytic leaves with a thicker leaf structure (Guerfel et al., 2009; Werden et al., 2017). Similarly, our results showed that coniferous species, especially *P. massoniana*, had the highest LT and the lowest LA and SLA in this community, which may be because it was the first pioneer tree species to enter this community; and in order to resist the arid and barren environment, they possessed leaf functional traits such as thicker leaves suitable for storing water and lower LA and SLA to reduce water loss through transpiration. This result was consistent with those of previous studies that showed larger SLA and thinner leaves of broad‐leaved trees than those of coniferous trees (Tian et al., 2016). Furthermore, the LN content of evergreen species was significantly lower than that of deciduous species because the generation cost of leaves was related to seasonal variation; leading to different adaptive strategies of evergreen and deciduous trees based on the variation of traits to adverse environments (Liu, Chen, et al., 2021; Liu, Li, et al., 2021). In summary, the variations in leaf functional traits in *L. glaber*–*C. glauca* evergreen broad‐leaved forest community were mainly attributed to the life form and interspecific variation, which was significantly affected by genetic background and taxon, and provided an important prerequisite for community assembly and species coexistence.

Meanwhile, there was a correlation between leaf structure and chemical traits in the community. SLA, LDMC, and LN all reflect adaptation strategies to the environment (Wright et al., 2004; Xun et al., 2020), and there was a significant positive correlation between SLA and LN, reflecting photosynthetic capacity and nutrient turnover at the species and community levels. SLA significantly correlated with LT, LDMC, and LC negatively, while no correlation was observed between LDMC and LT (Tables 4 and 5), indicating that LT and LDMC affected SLA in different ways in this community. Simultaneously, the construction of a leaf defense structure requires a large amount of photosynthate, and LC is usually used to compensate for consumption during development (Schulze et al., 1994). Therefore, LC increased with an increase in LDMC and LN. In addition, light is an important factor affecting SLA (Wyka et al., 2012), and LDMC is closely related to water (Saura‐Mas et al., 2009). The results showed that LDMC had lower interspecific variation and did not correlate with the chemical traits (except with LC), indicating that subtropical evergreen broad‐leaved forests had sufficient hydrothermal conditions and that the major factor affecting community assembly should be light rather than water.

### 4.2 Phylogenetic effects on leaf functional traits

The evolution is close to Brownian motion; that is, species with similar phylogenetic positions have similar characteristics and have certain evolutionary conservation. Species with similar functional traits are often phylogenetically similar (Losos, 2008). When a strong phylogenetic signal is detected in functional traits, environmental filters are probably selected for phylogenetically close species, causing phylogenetic clustering (Amaral et al., 2021). Here, only a number of leaf structural traits (LA and LT) showed strong and significant phylogenetic signals, indicating that LA and LT were closely related to phylogenetic history and showed strong phylogenetic conservation; that is, the more phylogenetically close species were more similar to LA and LT. All the dominant tree species had considerably high values of LA, except the deciduous tree species (*Q. fabri* and *A. kurzii* var. *Kurzii*). All evergreen tree species belonging to the same family and genus had more similar traits, especially for the two most dominant species (*L. glaber* and *C. glauca*) in the community, both belonging to Fagaceae (Table 2 and Figure 2).

The distributions of chemical traits (*K* < 1, *p* > .05; Figure 2) were not consistent with the phylogenetic relationships, indicating that the phylogenetic signals of these traits were random or divergent. Therefore, compared with the leaf chemical traits, the formation and development of structural traits were more affected by genetic differences, which is consistent with the results of Cao et al. (2013). In other words, the phylogenetic signal test based on functional traits showed a lack of consistency between the leaf functional trait patterns and phylogenetic patterns in this community, and no specific trend or relationship between them was observed. The phylogenetic relationships of *L. glaber*–*C. glauca* evergreen broad‐leaved forest community were inconsistent with the changes in functional traits with the historical processes. This observation was supported by the work of Cheng et al. (2019) on the construction mechanism of tropical cloud forest communities.

Numerous studies have indicated that phylogeny has a significant effect on the functional trait composition and that the relationships among traits are generally weakened after removing phylogeny (Cadotte et al., 2019; Liu, Chen, et al., 2021; Liu, Li, et al., 2021; Wang et al., 2020). This study also found that the traits of coexisting species in the community had a phylogenetic structure; however, only a few leaf traits (LA and LT) showed strongly phylogenetic signals. But the relationship among traits after removing the influence of the phylogeny remained a little changed at community level or even significantly enhanced at species level. This indicated that in the local community, environment had a greater impact on the spatial distribution of individuals, and plants follow the “realism” strategy to adapt to the environment, meaning, plants would adjust these trait‐off relationships according to their habitats to archive the best survival state, which was less limited by the evolutionary history. It was clear that the functional community structure was not consistent with the phylogenetic structure. Combined analysis of phylogenetic and functional trait structures will more accurately infer the main ecological processes driving species coexistence.

### 4.3 Community assembly mechanisms of integrated phylogenetic leaf functional traits

Many studies have attempted to distinguish between determinative and stochastic processes by partitioning the variation of species composition into environmental and spatial components (Chang et al., 2013; Legendre et al., 2009; Qiao et al., 2015). However, species niches are determined by their functional traits, which further influence their distribution along environmental gradients (McGill et al., 2006). Thus, the effects of determinative processes on community assembly are expected to be underestimated based on species identity, which does not consider the functional properties of species. However, we found that functional traits (12.35%) did not improve the interpretation rate of niche‐based processes by considering only species identity (28.10%, Zhao et al., 2015) and phylogenetic structure (29.80%; Figure 5). This observation was similar to that by Jiang et al. (2018) for temperate deciduous broad‐leaved Korean pine forests, in which functional traits could not better reveal ecological processes compared with that by species.

However, integrating functional traits with phylogeny can greatly improve the ability to infer determinative and stochastic processes, and trait‐ and phylogenetic‐based approaches are powerful ways to detect community assembly processes (Amaral et al., 2021; Li et al., 2019). We found that the interpretation rate of community assembly based on functional traits (63.38%) was higher than that based on phylogeny (47.96%). However, the pure space variable could significantly explain higher functional traits than that by environmental variables (Figure 5), indicating that the neutral stochastic process played a leading role in the construction of community functional traits. Compared with the community functional trait composition, the environment had a greater contribution to the spatial variation in the phylogenetic structure (Figure 5). This indicates that the phylogenetic structure of the community was aggregated (mainly affected by habitat filtration); however, leaf functional trait composition showed a dispersion pattern (mainly affected by stochastic processes). It also verified the opinion that species identity is a more holistic concept and could better depict multiple traits of a plant, while leaf functional traits could depict one or certain facets of a plant (Jiang et al., 2018). At the same time, among all edaphic and environmental variables, only altitude, aspect, and TK reflecting light and water utilization of plants had a significant effect on community functional composition (Figure 4), which was related to the fact that leaves were key organs of plant photosynthesis and mainly exercise the functions of photosynthesis and nutrient turnover. Therefore, we need to consider more functional traits (such as height and wood density) to provide more detailed information to improve the interpretation rate of community functional trait composition.

## 5 CONCLUSIONS

The phylogenetic and trait‐based analyses conducted in this study showed that functional traits and phylogeny provide a meaningful way to detect community assembly processes. The results show that there were significant interspecific variations in leaf traits at *L. glaber*–*C. glauca* evergreen broad‐leaved forest community. Leaf functional trait composition of coexisting species showed dispersion pattern and tended to adopt “realism” to adapt to their habitats. Strong phylogenetic signals were detected only in LA and LT. In addition, TK, TP, altitude, aspect, and convexity were the primary influencing environmental factors. *Neutral‐theory‐based stochastic processes* were the main drivers in the development of community leaf functional traits, but niche‐based habitat filtration was more important influencing mechanism of community phylogenetic structure. In future research, more functional traits as well as a larger scale should be taken into account.

## AUTHOR CONTRIBUTIONS

**Lijuan Zhao:** Conceptualization (equal); data curation (equal); formal analysis (lead); funding acquisition (lead); investigation (lead); methodology (lead); resources (equal); writing – original draft (equal); writing – review and editing (equal). **Wenhua Xiang:** Conceptualization (supporting); methodology (equal); resources (supporting); writing – review and editing (equal). **Jiaxiang Li:** Conceptualization (equal); data curation (lead); formal analysis (equal); investigation (lead); methodology (equal); software (equal); writing – original draft (equal); writing – review and editing (equal). **Wenqian Liu:** Data curation (supporting); investigation (equal); methodology (equal); software (supporting); writing – original draft (equal). **Yanting Hu:** Conceptualization (supporting); methodology (supporting); writing – review and editing (equal). **Huili Wu:** Data curation (supporting); methodology (supporting); writing – original draft (equal). **Yiling Zhang:** Data curation (supporting); investigation (equal); validation (supporting). **Xing Cheng:** Data curation (supporting); formal analysis (supporting); investigation (equal); methodology (equal); writing – original draft (equal). **Weijia Wang:** Data curation (supporting); investigation (equal); writing – original draft (supporting). **Wentao Wang:** Investigation (supporting); software (supporting). **Shuai Ouyang:** Conceptualization (equal); methodology (supporting); writing – original draft (equal); writing – review and editing (equal).

## FUNDING INFORMATION

National Natural Science Foundation of China, Grant/Award Number: 31901136. Undergraduate Training Program for Innovation and Entrepreneurship of Hunan Province, Grant/Award Number: S202210538073.

## Data Availability

All input data and scripts for each analysis can be found in a permanent Dryad repository https://doi.org/10.5061/dryad.2jm63xss6.
