# Corrigendum

**DOI:** 10.1002/ece3.9392

**Published:** 2022-10-03

In the recent article by Zhao et al. (2022), the map of China in figure 1 was incomplete. The correct figure is shown below:

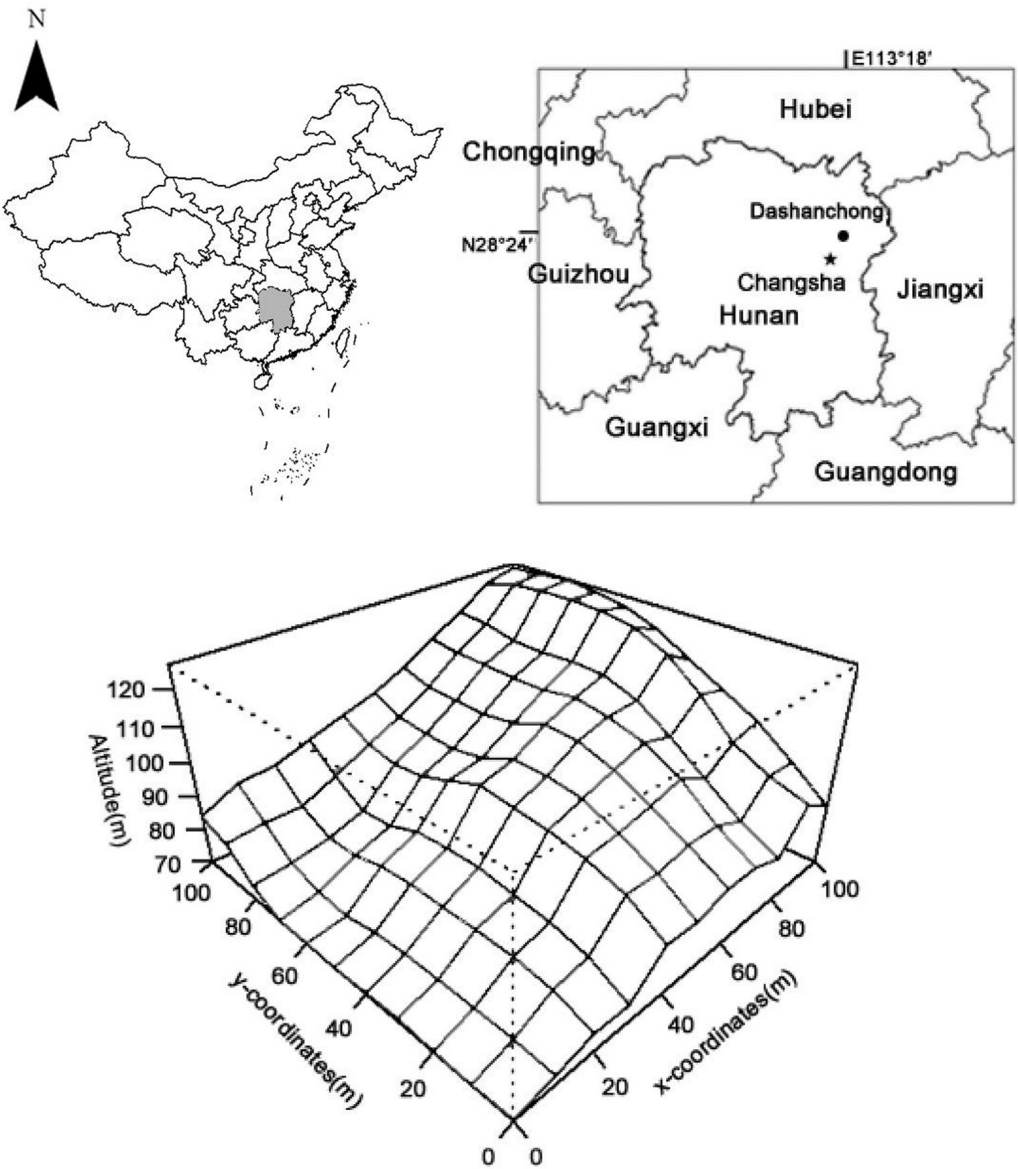

The authors apologize for the error.
